# Mechanisms, molecular and sero-epidemiology of antimicrobial resistance in bacterial respiratory pathogens isolated from Japanese children

**DOI:** 10.1186/1476-0711-6-7

**Published:** 2007-08-13

**Authors:** Keisuke Sunakawa, David J Farrell

**Affiliations:** 1 School of Medicine, Kitasato University, Kanagawa, Japan; 2 G.R. Micro Ltd, London, UK

## Abstract

**Background:**

The clinical management of community-acquired respiratory tract infections (RTIs) is complicated by the increasing worldwide prevalence of antibacterial resistance, in particular, β-lactam and macrolide resistance, among the most common causative bacterial pathogens. This study aimed to determine the mechanisms and molecular- and sero-epidemiology of antibacterial resistance among the key paediatric respiratory pathogens in Japan.

**Methods:**

Isolates were collected at 18 centres in Japan during 2002 and 2003 from children with RTIs as part of the PROTEKT surveillance programme. A proportion of *Haemophilus influenzae *isolates was subjected to sequencing analysis of the *ftsI *gene; phylogenetic relatedness was assessed using multilocus sequence typing. *Streptococcus pneumoniae *isolates were screened for macrolide-resistance genotype by polymerase chain reaction and serotyped using the capsular swelling method. Susceptibility of isolates to selected antibacterials was performed using CLSI methodology.

**Results and Discussion:**

Of the 557 *H. influenzae *isolates collected, 30 (5.4%) were β-lactamase-positive [BL+], 115 (20.6%) were BL-nonproducing ampicillin-resistant (BLNAR; MIC ≥ 4 mg/L) and 79 (14.2%) were BL-nonproducing ampicillin-intermediate (BLNAI; MIC 2 mg/L). Dabernat Group III penicillin binding protein 3 (PBP3) amino acid substitutions in the *ftsI *gene were closely correlated with BLNAR status but phylogenetic analysis indicated marked clonal diversity. PBP mutations were also found among BL+ and BL-nonproducing ampicillin-sensitive isolates. Of the antibacterials tested, azithromycin and telithromycin were the most active against *H. influenzae *(100% and 99.3% susceptibility, respectively). A large proportion (75.2%) of the 468 *S. pneumoniae *isolates exhibited macrolide resistance (erythromycin MIC ≥ 1 mg/L); *erm*(B) was the most common macrolide resistance genotype (58.8%), followed by *mef*(A) (37.2%). The most common pneumococcal serotypes were 6B (19.7%), 19F (13.7%), 23F (13.5%) and 6A (12.8%). Telithromycin and amoxicillin-clavulanate were the most active antibacterials against *S. pneumoniae *(99.8% and 99.6% susceptibility, respectively).

**Conclusion:**

Approximately one-third of *H. influenzae *isolates from paediatric patients in Japan are BLNAI/BLNAR, mainly as a result of clonally diverse PBP3 mutations. Together with the continued high prevalence of pneumococcal macrolide resistance, these results may have implications for the clinical management of paediatric RTIs in Japan.

## Background

Community-acquired respiratory tract infections (RTIs) such as pneumonia, bronchitis, tonsillitis/pharyngitis, otitis media and bacterial sinusitis are prevalent conditions accounting for approximately three-quarters of all outpatient antibacterial prescriptions [1], a large proportion of which are for children. Antibacterial therapy for community-acquired RTIs is usually empirical [2]. However, the clinical management of community-acquired RTIs is complicated by the increasing worldwide prevalence of antibacterial resistance, in particular β-lactam and macrolide resistance, among the most common causative bacterial pathogens [3-7]. This has important implications for the selection of appropriate and effective antibacterial therapy, especially for children in whom current treatment options are largely limited to these two classes of antibacterial agents.

*Haemophilus influenzae *is frequently implicated in paediatric community-acquired RTIs. Resistance of *H. influenzae *to ampicillin has been increasing steadily since its emergence in the 1970s [8]. Until recently, β-lactamase (BL) production has been the primary mechanism of ampicillin resistance among *H. influenzae*. However, the prevalence of BL-nonproducing ampicillin-resistant (BLNAR) strains of *H. influenzae *now also appears to be increasing [9,10]. This is of clinical significance, since BLNAR *H. influenzae *are typically co-resistant to other commonly prescribed β-lactams, including amoxicillin-clavulanate and ampicillin-sulbactam, in addition to most cephalosporins [11].

The increasing global prevalence of antibacterial resistance among *Streptococcus pneumoniae *– another significant respiratory pathogen – is cause for further concern. As a result of the rapid evolution of β-lactam-resistant *S. pneumoniae*, macrolides have been increasingly used as initial empirical therapy in community-acquired RTIs [2]. However, the global increase in macrolide-resistant strains of *S. pneumoniae *now also threatens to compromise the use of these antibacterials for the treatment of these conditions [4].

Such resistance trends highlight an urgent need for new antibacterials for the treatment of paediatric community-acquired RTIs that are effective against the common respiratory pathogens, but which retain activity against isolates resistant to current treatment options.

PROTEKT (**P**rospective **R**esistant **O**rganism **T**racking and **E**pidemiology for the **K**etolide **T**elithromycin) is a global, multicentre surveillance study investigating the antimicrobial susceptibility of bacterial pathogens associated with community-acquired RTIs. As part of this survey, a study was undertaken in Japan to assess the BL status of *H. influenzae *isolates collected from paediatric patients with community-acquired RTIs and to determine the mechanism of resistance and molecular epidemiology of BLNAR strains. The activities of several antibacterial agents against these isolates and other key respiratory pathogens were also assessed.

## Methods

### Isolate collection

Bacterial isolates were collected over four 1-week periods (24–30 November 2002; 19–26 January 2003; 9–15 March 2003; and 15–21 June 2003) from children (aged < 16 years) with community-acquired RTIs (sinusitis, tonsillitis, pharyngitis/laryngitis, otitis media, pneumonia, bronchitis and others) at 18 centres in Japan. Acceptable sources for isolates included blood, sputum, bronchoalveolar lavage fluid, middle-ear fluid, nasopharyngeal swabs or aspirates, sinus aspirates and throat swabs. Isolates from patients with nosocomial RTIs or cystic fibrosis were excluded from this analysis. Duplicate strains, isolates originating from existing collections and those of doubtful pathogenicity were also excluded.

### Susceptibility testing

All isolates were transported to a central laboratory for susceptibility testing (Mitsubishi Kagaku Bio-Clinical Laboratories Inc., Japan). For *H. influenzae*, BL production was detected using the chromogenic nitrocefin method (Unipath Ltd, Basingstoke, UK) and by isolates being nonsusceptible to ampicillin MIC ≥ 2 mg/L). MICs of isolates to a panel of antibacterial agents (including telithromycin, erythromycin, azithromycin, ampicillin, amoxicillin-clavulanate and cefdinir) were determined using the Clinical and Laboratory Standards Institute (CLSI) broth microdilution method [11] and interpreted using established CLSI breakpoints [12].

### Molecular methods

A proportion of *H. influenzae *isolates were analysed by polymerase chain reaction (PCR) amplification and sequencing of regions of the *ftsI *gene encoding the transpeptidase domain of penicillin-binding protein (PBP) 3A and/or PBP 3B as described by Dabernat et al. [13] Phylogenetic relationships among *H. influenzae *strains were determined by multilocus sequence typing (MLST) of seven housekeeping genes as described previously [14].

All isolates of *S. pneumoniae *found to be resistant to erythromycin (MIC ≥ 1 mg/L) were analysed for the presence of *erm*(B), *mef*(A) and *erm*(A) subclass *erm*(TR) macrolide resistance gene sequences using a rapid-cycle multiplex PCR method as described previously [15].

Serotyping of *S. pneumoniae *isolates was performed at G.R. Micro Ltd (London, UK) using the Neufeld's quellung reaction with Statens Serum Institute (SSI) antisera (SSI, Copenhagen, Denmark). The SSI was used as the reference laboratory for quality assurance and rare serotypes.

## Results

### Patients

A total of 5,592 patients were included in this analysis. Key demographics and patient characteristics are summarized in Table 1. Almost two-thirds (64.5%) of study participants were aged 0–5 years. Upper RTIs accounted for 61.6% of all community-acquired RTIs reported. Pharyngitis/laryngitis was the most common upper RTI (33.5%), followed by unspecified upper RTIs and tonsillitis (14.1% and 7.1%, respectively). Bronchitis was the most common lower RTI, occurring in 23.4% of patients. Just over half of all study participants (53.3%) received antibacterial therapy for the treatment of their community-acquired RTI.

**Table 1:** Key demographics and patient characteristics (combined data for all four 1-week study periods).

	**Number (%) of patients (n = 5592)**
**Sex**	
Male	2942 (52.6)
Female	2625 (46.9)
Unknown	25 (0.4)
	
**Age (years)**	
0–2	2245 (40.1)
>2–5	1363 (24.4)
>5–10	1557 (27.8)
>10–16	427 (7.6)
	
**Type of RTI^1^**	
Pneumonia	154 (2.7)
Bronchitis	1350 (23.4)
Pharyngitis/laryngitis	1933 (33.5)
Tonsillitis	409 (7.1)
Sinusitis	140 (2.4)
Otitis media	146 (2.5)
Unspecified upper RTI	814 (14.1)
Other^2^	826 (14.3)
Unknown	2 (0.03)
	
**Antibacterial therapy**	
Present	2981 (53.3)
Absent	2611 (46.7)

^1^More than one RTI reported in some patients. Percentages are calculated using total number of RTIs (n = 5774).

^2^Other infections: acroposthitis; acute gastroenteritis; acute pneumonia; acute rhinitis; adenovirus infection; allergic bronchitis; asthmatic bronchitis; bronchiolitis; allergic/asthmatic bronchitis; cervical lymphadenitis; chlamydial infection; conjunctivitis; cough; enzootic fever; epidemic catarrh; erythema infectiosum; exanthema subitum; haemolytic streptococcal infection; hand, foot and mouth disease; herpangina; lacunar tonsillitis; lateral pharyngitis; mycoplasma infection; mycoplasma pneumonia; parotitis; pertussis; pseudocroup; rhinitis; suppurative tonsillitis; sinobronchitis; tuberculosis; viral infection.

RTI, respiratory tract infection.

### Bacterial isolates

A total of 2,596 pathogens were collected in this study, including 557 isolates of *H. influenzae *and 468 isolates of *S. pneumoniae*.

#### Haemophilus influenzae

Of the 557 *H. influenzae *isolates collected, 115 (20.6%) were BLNAR (MIC ≥ 4 mg/L) and 79(14.2%) were BL-nonproducing ampicillin-intermediate (BLNAI MIC 2 mg/L). In total, 30(5.4%) isolates were found to be BL+.

**Figure 1:**
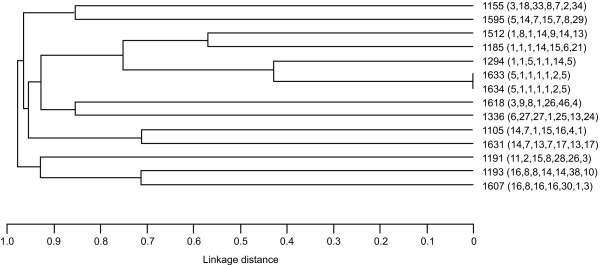
Phylogenetic relationships based on sequence-type variations found in *Haemophilus influenzae *that were β-lactamase nonproducing ampicillin-resistant with β-lactamase nonproducing ampicillin-sensitive (BLNAS; n = 14).

**Figure 2:**
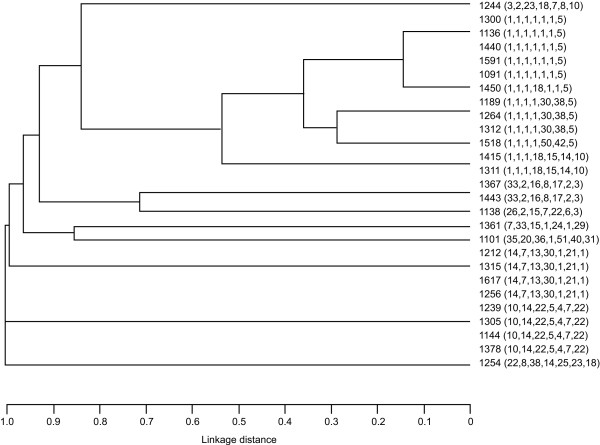
Phylogenetic relationships based on sequence-type variations found in *Haemophilus influenzae *that were β-lactamase nonproducing ampicillin-resistant with βlactamase positive (BL+; n = 27).

A total of 110 BLNAR isolates were viable for molecular analysis and a further 5 BLNAI, 14 BL-nonproducing ampicillin-sensitive (BLNAS), and 27 BL+ isolates were examined for comparison. In the 156 isolates analysed, *ftsI *mutations resulted in amino acid substitutions at 23 different loci and 36 different combinations of these substitutions were found. One particular combination of substitutions predominated (D350N, S357N, M377I, S385T, L389F, N526K) – corresponding to PBP 3 Group III in the classification scheme described by Dabernat et al. [13], accounting for 58.3% (91/156) of the isolates (Table 2). Classification of the remaining isolates into Dabernat groups was difficult owing to the large number of previously undescribed mutations. The distribution of amino acid substitution types according to β-lactamase and ampicillin resistance status is shown in Table 2. Of the BLNAR isolates, 75.5% (83/110) were classified as Dabernat Group III. None of the BLNAR or BLNAI isolates had the wild-type (i.e., ampicillin-susceptible) PBP 3 sequence. By contrast, 39.0% (16/41) of the isolates that were either BLNAS or BL+ had the wild-type PBP 3 sequence, with the remainder having mainly non-Group III substitution patterns. MLST of the 156 isolates identified a total of 73 different sequence types: this lack of genetic similarity in the BLNAR PBP 3 Group III, BLNAS and BL+ *H. influenzae *isolates is shown in Additional file 1 and Figures 1, 2.

**Table 2:** Distribution of β-lactamase and ampicillin status by penicillin binding protein 3 substitution grouping [13] for 156 *Haemophilus influenzae *isolates.

**Phenotype**	**n**	**Group III**	**Other groups**	**Wild type**
BLNAR	110	83	27	0
BLNAI	5	3	2	0
BLNAS	14	1	6	7
BL+	27	4	14	9

BL+, β-lactamase positive; BLNAI, β-lactamase-nonproducing ampicillin-intermediate; BLNAR, β-lactamase-nonproducing ampicillin-resistant; BLNAS, β-lactamase-nonproducing ampicillin-sensitive.

Azithromycin and telithromycin both showed good *in vitro *activity against *H. influenzae *isolates (Table 3). All 557 *H. influenzae *isolates were susceptible to azithromycin (MIC ≤ 4 mg/L), while 553/557 (99.3%) were found to be fully susceptible to telithromycin (MIC ≤ 4 mg/L), including 99.5% (193/194) of BLNAR/I and 96.7% (29/30) of BL+ isolates. The remaining four isolates (0.7%) exhibited intermediate susceptibility to telithromycin (MIC 8 mg/L). The majority of *H. influenzae *isolates, 81.7% (455/557), were susceptible to amoxicillin-clavulanate (MIC ≤ 4 mg/L); 59.8% (333/557) were susceptible to ampicillin (MIC ≤ 1 mg/L); and 63.9% (356/557) were susceptible to cefdinir (MIC ≤ 1 mg/L) (Table 3).

**Table 3:** Activity of antibacterial agents against 557 isolates of *H. influenzae *collected from Japanese children (aged < 16 years) with community-acquired respiratory tract infections (combined data for all four 1-week study periods).

	**MIC (mg/L)**					
		
**Antibacterial**	**Mode**	**MIC_50_**	**MIC_90_**	**Range**	**Susceptibility breakpoint**	**% susceptibility**
Telithromycin	2	2	4	≤0.06–8	≤4	99.3
Erythromycin	8	8	8	0.5–16	NA	NA
Azithromycin	2	2	2	≤0.06–4	≤4	100.0
Cefdinir	0.5	0.5	8	≤0.06–32	≤1	63.9
Ampicillin	0.5	0.5	8	≤0.06–>128	≤1	59.8
Amoxicillin-clavulanate	0.5	1	8	≤0.06–32	≤4	81.7

NA, not available.

#### Streptococcus pneumoniae

Genotyping analysis of the 468 isolates of *S. pneumoniae *collected during this study is shown in Table 4. Of the 352 isolates (75.2%) found to be macrolide-resistant (erythromycin MIC ≥ 1 mg/L), the vast majority expressed either the *erm*(B) (58.8%) or *mef*(A) (37.2%) macrolide resistance genes. A small number of erythromycin-sensitive or -intermediate *S. pneumoniae *isolates also expressed *erm*(B) or *mef*(A).

**Table 4:** Genotyping analysis by erythromycin resistance phenotype of *Streptococcus pneumoniae *isolates (n = 468) collected from Japanese children (aged < 16 years) with community-acquired respiratory tract infections (combined data for all four 1-week study periods).

	**No. of isolates (%)**		
	
**Genotype**	**ERY-S (n = 104)**	**ERY-I (n = 12)**	**ERY-R (n = 352)**
*erm*(B)	5 (4.8)	3 (25.0)	207 (58.8)
*erm*(B) + *mef*(A)	1 (1.0)	0 (0.0)	8 (2.3)
*mef*(A)	1 (1.0)	7 (58.3)	131 (37.2)
*erm*(A) subclass *erm*(TR)	0 (0.0)	0 (0.0)	1 (0.3)
Negative for mechanisms tested	97 (93.3)	1 (8.3)	1 (0.3)
Not viable for testing	0 (0.0)	1 (8.3)	4 (1.1)

ERY-I, erythromycin-intermediate (MIC 0.5 mg/L); ERY-R, erythromycin-resistant(MIC ≥ 1 mg/L); ERY-S, erythromycin-sensitive (MIC ≤ 0.25 mg/L).

**Figure 3:**
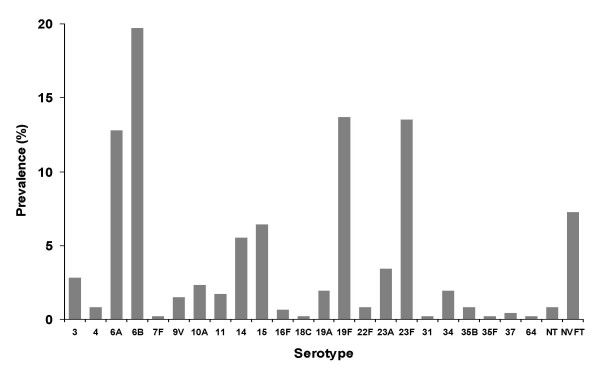
Serotype distribution of 468 isolates of *Streptococcus pneumoniae *collected from Japanese children (aged < 16 years) with community-acquired respiratory tract infections (combined data for all four 1-week study periods).

The results of the serotyping analysis of all 468 *S. pneumoniae *isolates are shown in Figure 3. Overall, the most common serotypes were 6B (92/468 [19.7%]), 19F (64/468 [13.7%]), 23F(63/468 [13.5%]) and 6A (60/468 [12.8%]). The serotype distributions for erythromycin-resistant, -intermediate and -sensitive isolates were similar.

The *S. pneumoniae in vitro *susceptibility rate for telithromycin (99.8% [467/468]; the remaining oneisolate had intermediate susceptibility [MIC 2 mg/L]) and was comparable to that observed for amoxicillin-clavulanate (99.6%). Lower susceptibility rates were recorded for ampicillin (93.4%), erythromycin (22.2%), azithromycin (24.6%) and cefdinir (58.8%) (Table 5).

**Table 5:** Activity of antibacterial agents against 468 isolates of *Streptococcus pneumoniae *collected from Japanese children (aged < 16 years) with community-acquired respiratory tract infections (combined data for all four 1-week study periods).

	**MIC (mg/L)**					
		
**Antibacterial**	**Mode**	**MIC_50_**	**MIC_90_**	**Range**	**Susceptibility breakpoint**	**% susceptibility**
Telithromycin	≤0.06	≤0.06	0.12	≤0.06–2	≤1	99.8
Erythromycin	>128	4	>128	≤0.06–>128	≤0.25	22.2
Azithromycin	>16	4	>16	≤0.06–>16	≤0.5	24.6
Cefdinir	4	1	8	≤0.06–32	≤2	58.8
Ampicillin	≤0.06	0.5	2	≤0.06–8	≤2	93.4
Amoxicillin-clavulanate	≤0.06	0.25	1	≤0.06–4	≤2	99.6

## Discussion

Results of this study indicate that a high prevalence of *in vitro *resistance to conventional antibacterial agents, such as azithromycin, erythromycin and cefdinir, existed among the key paediatric respiratory pathogens in Japan during the surveillance period in 2002 and 2003. Although the data cannot be directly extrapolated to the current status of antibacterial resistance in Japan, the study highlights a number of potentially important issues regarding the antibacterial susceptibility and epidemiology of the key respiratory tract pathogens.

One-fifth of all isolates of *H. influenzae *were found to be BLNAR (20.6%), with a further 14.2% BLNAI and 5.4% BL+. This is in keeping with the findings of other Japanese studies, with results of one recent nationwide survey showing 23.1% of all *H. influenzae *isolates collected to be BLNAR and 6.0% BL+ [16]. Molecular epidemiological analysis of a proportion of the *H. influenzae *isolates collected in this study indicated that Dabernat Group III PBP 3 amino acid substitutions in the *ftsI *gene were found to correlate highly with BLNAR status. However, there was marked clonal diversity among all *H. influenzae *isolates, suggesting that the *ftsI *mutations associated with BLNAR status have developed independently in different strains at a relatively high frequency, rather than through clonal expansion of a successful strain. Mutations in *ftsI *were also seen in more than half of the BLNAS and BL+ isolates analysed. BL+ *H. influenzae *strains exhibiting resistance to amoxicillin-clavulanate and/or cephalosporin antibiotics have been identified previously in isolates collected in various countries, including Japan [17,18]. The identification of *ftsI *mutations in some BL+ *H. influenzae *strains [11,19,20] may, in some cases, explain their decreased susceptibility to non-β-lactam antibiotics.

Both azithromycin and telithromycin demonstrated good *in vitro *activity against *H. influenzae *isolates collected in this study, irrespective of BL+ or BLNAR/I, resistance mechanism or serotype status. In contrast, only 24.6% of *S. pneumoniae *isolates were fully susceptible to azithromycin, while telithromycin was highly active against this pathogen, including isolates resistant to macrolides. Overall, three-quarters of the 468 *S. pneumoniae *isolates were found to be macrolide-resistant, whereas only 1 isolate exhibited low-level resistance to telithromycin. These observations are in keeping with the results of previous analyses of paediatric *S. pneumoniae *isolates, which showed telithromycin to be highly active against all strains, irrespective of macrolide, azalide or clindamycin resistance status [21,22].

To date, no conjugate pneumococcal vaccine has been routinely used in Japan. The 7-valent conjugate vaccine (PCV-7) is licensed in the USA and Europe and has coverage against serotypes 4, 6B, 9V, 14, 18C, 19F and 23F. Only 55.2% (257/465) of the isolates in this study were serotypes covered by PCV-7. If the potentially cross-reacting serotypes (6A and 19A) were added, coverage was raised to 70.1%. However, the uncertainty regarding cross-protection and the potential for serotype replacement underscore the need for serotype surveillance [23].

Although the results presented in this paper may have important implications for the empiral antibiotic treatment of paediatric RTIs in Japan, two factors limit the degree to which the data can be interpreted. Firstly, no attempt was made to correlate *in vitro *resistance with resistance *in vivo *that may lead to adverse clinical outcome. Secondly, the study did not distinguish between isolates that may be colonising the respiratory tract from those actually causing the infection. This is particularly important when considering paediatric RTIs, as carriage of common respiratory tract pathogens, such as *S. pneumoniae*, in children is extremely common [24].

## Conclusion

In conclusion, this study provides a snapshot of the antibacterial susceptibility and epidemiology of key respiratory tract pathogens isolated from children in Japan during 2002 and 2003. Approximately one-third of *H. influenzae *isolates were BLNAI/BLNAR, mainly as a result of clonally diverse PBP3 mutations. PBP3 mutations were also common among BL+ and BLNAS isolates. Together with the observed high prevalence of *in vitro *pneumococcal macrolide resistance, these results may have implications for the clinical management of paediatric RTIs in Japan.

## Competing interests

KS has no competing interests to declare.

DJF has received research grants and consultancy fees from sanofi-aventis related to telithromycin research, publications and presentations.

## Authors' contributions

KS has made substantial contributions to conception and design and acquisition of data, and drafted the manuscript.

DJF and colleagues at GR Micro Limited undertook the laboratory testing, data collection and analysis, and drafted the paper.

Both authors have read and approved the final manuscript.

## Supplementary Material

Additional file 1 Phylogenetic relationships. Phylogenetic relationships based on sequence-type variations found in *Haemophilus influenzae *that were β-lactamase nonproducing ampicillin-resistant with Group III PBP 3 mutations (BLNAR Group III; n = 83) Click here for file
