# Supplementary figures and images for: Mechanisms, molecular and sero-epidemiology of antimicrobial resistance in bacterial respiratory pathogens isolated from Japanese children
Source: Ann Clin Microbiol Antimicrob. 2007 Aug 13;6:7. doi: 10.1186/1476-0711-6-7 (PMC2020463; doi:10.1186/1476-0711-6-7)

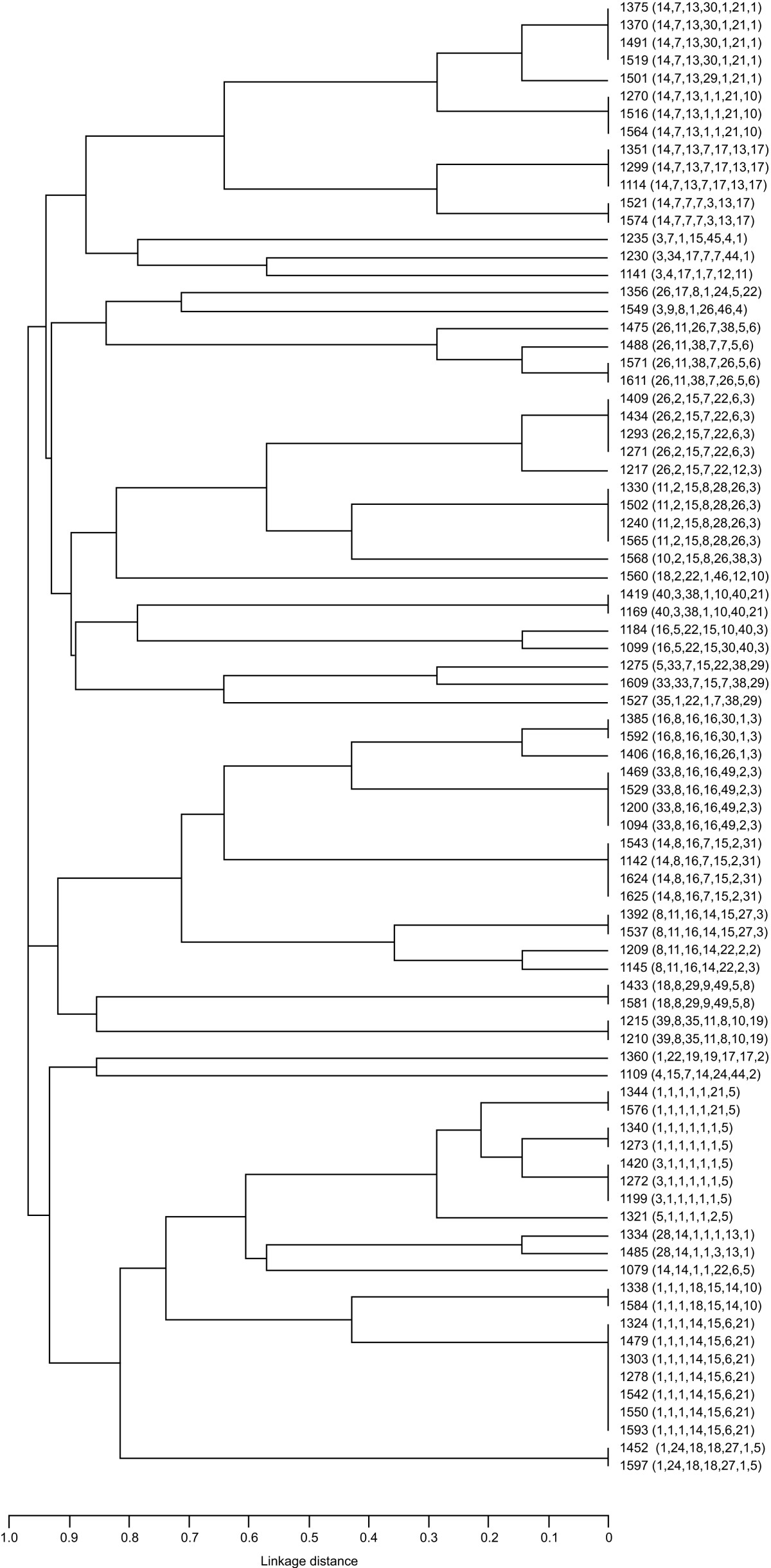

Supplement: Additional file 1 — Phylogenetic relationships. Phylogenetic relationships based on sequence-type variations found in Haemophilus influenzae that were β-lactamase nonproducing ampicillin-resistant with Group III PBP 3 mutations (BLNAR Group III; n = 83) [file 1476-0711-6-7-S1.jpeg]
